# Functional Genome Analyses Reveal the Molecular Basis of Oil Accumulation in Developing Seeds of Castor Beans

**DOI:** 10.3390/ijms25010092

**Published:** 2023-12-20

**Authors:** Anmin Yu, Zekun Zhou, Yizhong Chen, Jing Sun, Ping Li, Xu Gu, Aizhong Liu

**Affiliations:** Key Laboratory for Forest Resources Conservation and Utilization in the Southwest Mountains of China, Ministry of Education, Southwest Forestry University, Kunming 650224, China; anminyu@swfu.edu.cn (A.Y.); zhouzk2023@163.com (Z.Z.); 13108653987@163.com (Y.C.); 18214636754@163.com (J.S.); liping2020@swfu.edu.cn (P.L.); guxu675129144@163.com (X.G.)

**Keywords:** castor, ricinoleic acid, seed, gene expressions

## Abstract

Castor (*Ricinus communis* L.) seeds produce abundant ricinoleic acid during seed maturation, which is important for plant development and human demands. Ricinoleic acid, as a unique hydroxy fatty acid (HFA), possesses a distinct bond structure that could be used as a substitute for fossil fuels. Here, we identified all homologous genes related to glycolysis, hydroxy fatty acid biosynthesis, and triacylglycerol (TAG) accumulation in castor seeds. Furthermore, we investigated their expression patterns globally during five seed development stages. We characterized a total of 66 genes involved in the glycolysis pathway, with the majority exhibiting higher expression levels during the early stage of castor bean seed development. This metabolic process provided abundant acetyl-CoA for fatty acid (FA) biosynthesis. Subsequently, we identified 82 genes involved in the processes of de novo FA biosynthesis and TAG assembly, with the majority exhibiting high expression levels during the middle or late stages. In addition, we examined the expression patterns of the transcription factors involved in carbohydrate and oil metabolism. For instance, *RcMYB73* and *RcERF72* exhibited high expression levels during the early stage, whereas *RcWRI1*, *RcABI3*, and *RcbZIP67* showed relatively higher expression levels during the middle and late stages, indicating their crucial roles in seed development and oil accumulation. Our study suggests that the high HFA production in castor seeds is attributed to the interaction of multiple genes from sugar transportation to lipid droplet packaging. Therefore, this research comprehensively characterizes all the genes related to glycolysis, fatty acid biosynthesis, and triacylglycerol (TAG) accumulation in the castor and provides novel insight into exploring the genetic mechanisms underlying seed oil accumulation in the endosperm of castor beans.

## 1. Introduction

Biodiesel is a renewable and clean-burning fuel, which is necessary for environmental and economic sustainability. As a potential substitute for fossil fuels, biodiesel possesses properties that are comparable to or better than petro-diesel. Notably, it contributes to reduced CO_2_ and exhaust emissions, as well as improved combustion efficiency [1]. Predominantly, biodiesel is derived from natural and renewable sources, such as vegetable oil, waste animal fats, and oils [2]. However, due to certain inherent disadvantages in biodiesel production, such as higher costs, increased density, and greater viscosity, the industrialization of biodiesel production has faced great challenges in recent years [3]. Despite various efforts to utilize vegetable oils, such as rapeseed oils, soybean oils, jatropha oils, castor oils, and rubber seed oils, for biodiesel production, controversies still exist. The increasing demand for edible vegetable oils has posed challenges to the utilization of these oils for biodiesel production, as this may disturb the edible vegetable oil market [4]. However, utilizing non-edible vegetable oils, such as castor and jatropha seed oils, appears to be a favorable and acceptable approach for biodiesel production [5]. In particular, castor oil is highly regarded as an excellent biodiesel feedstock due to its complete solubility with ethanol at any proportion [6].

Castor oil, derived from the seeds of the castor bean (*Ricinus communis* L., 2n = 20), is distinctive among vegetable oils due to its high content of hydroxy fatty acid (ricinoleic acid, 18C:1-OH), which exceeds 90%. This unique biochemical profile has established castor oil as a valuable feedstock for a wide array of chemical products, including lubricants, paints, inks, nylon, and cosmetics [7]. The castor bean is widely cultivated, particularly in India, Brazil, and China, attributed to its high seed oil content, which can constitute up to 50% of the seed’s dry weight, and its significant economic value [8]. Furthermore, due to its growth characteristics, such as a short gestation period, drought tolerance, and adaptability to diverse soil conditions, the castor bean has garnered significant attention worldwide for its potential to utilize marginal lands for the commercial production of its seed oil as s source of industrial oils and biodiesel [9]. However, despite years of domestication, many high-yield castor bean varieties cultivated on marginal lands frequently exhibit inconsistent seed oil content, leading to diminished agricultural economic returns. There is an urgent need for the genetic enhancement of castor bean varieties to develop improved strains that can better serve agricultural applications. Understanding the molecular basis of oil accumulation during seed development is an essential prerequisite for understanding the genetic factors that regulate lipid biosynthesis in castor seeds and for creating improved varieties through genetic breeding.

In general, the pathway of storage lipid (in the form of triacylglycerol, abbreviated TAG) biosynthesis in higher plants has been documented, mainly including two conceptually simplified systems: fatty acid (FA) de novo synthesis in plasmids and TAG assembly in the endoplasmic reticulum (ER) [10]. The FA de novo synthesis produces a common and unique system intermediate, cytosolic acyl-CoA. The TAG assembly process utilizes the acyl-CoA produced in the previous step, incorporating it sequentially with glycerol-3-phosphate (G-3-P) in the ER [11]. The quantitative oil content and fatty acid composition in developing seeds are regulated by multiple enzyme genes and regulators. While the functions of many individual enzyme genes and regulators involved in the oil biosynthesis pathway are well documented, there is limited knowledge about the overall expression and regulation profiles of the multiple genes involved in de novo carbon flux to TAG during seed development [12]. This aspect of storage lipid accumulation is important for the attempts to identify the key rate-limiting enzyme genes and regulators in developing oilseeds. In contrast to photosynthetic oilseed crops, such as rapeseeds and soybeans, castor bean seeds are typically non-photosynthetic. The carbon sources required for oil biosynthesis in developing seeds rely entirely on photosynthesis and glycolysis products in leaves, as well as the transport of sugar through the phloem from the leaves to developing seeds [13]. Specifically, the biosynthesis of the unique ricinoleic acid oils involves an independent phospholipid-diacylglycerol acyltransferase (PDAT) pathway during TAG assembly in developing castor seeds [14]. The castor bean is often considered a model system to dissect the molecular basis of oil accumulation in developing seeds.

With recent updates in the castor reference genome and the expanded transcriptome data pertaining to seed development [15,16], a more nuanced understanding of the gene expression and regulation involved in the de novo carbon flux to triacylglycerol (TAG) in developing seeds has been achieved. This advancement facilitates the identification of the critical rate-limiting enzymes and regulatory genes that are likely instrumental in controlling oil accumulation in developing castor oilseeds. Based on comprehensive gene co-expression analysis and the integration of various pathways related to oil biosynthesis, this study provides a global molecular network profile of oil accumulation in developing castor seeds and establishes a foundation for exploring the genetic mechanisms underlying oil accumulation and potential pathways for breeding improvement.

## 2. Results

### 2.1. Dynamics of Oil Accumulation in Developing Seeds of Castor Beans

**Figure 1:**
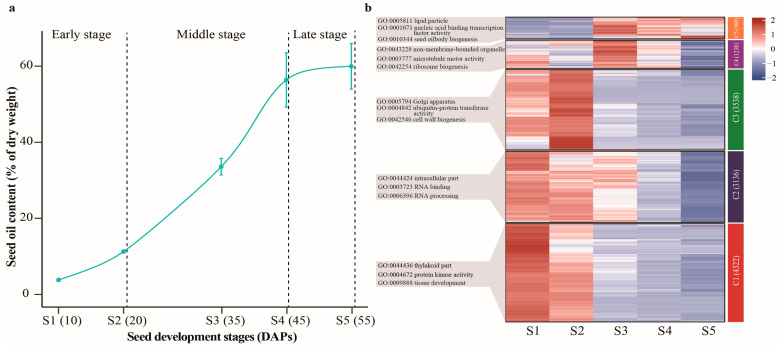
Characterization of castor seed oil and transcriptomic characteristics during seed development. (**a**) The oil content during castor seed development process. The whole seed development process of castor variety ZB306 was divided into three stages, the early (10–20 DAP, days after pollination), middle (21–45 DAP), and late stage (46–55 DAP). (**b**) Heatmap and hierarchical clustering of genes involved in five seed developmental stages. The colors represent the relative gene expression values after normalization adjustments. The red and blue colors refer to up- and down-regulation, respectively.

While the duration of seed development and oil content (TAG content) varied among different cultivars, previous investigations have generally characterized the seed development process of castor beans into three typical stages: the early stage, the rapid oil accumulation stage, and the late stage [17,18]. To characterize the process of oil accumulation during seed development, we divided the seed development of variety ZB306 into three stages, the early (10–20 DAP, days after pollination), middle (21–45 DAP), and late stage (46–55 DAP), as shown in Figure 1a. Physiologically, in the early stage, there is the down-loading of soluble sugar from capsules where basic carbohydrate metabolism occurs, although the accumulation of TAG does not begin. In the middle stages, there is intensive biosynthesis of storage materials (such as storage lipids and proteins) in conjunction with the rapid increase in seed volume and weight. In the late stage, the accumulation rate of biomass metabolism slows down and seed dehydration occurs (Figure 1a). In particular, during the middle stage of castor seed development (Figure 1), oil biosynthesis undergoes initial, rapid, and decelerated accumulation processes, indicating the finely regulated nature of TAG biosynthesis. In order to elucidate the physiological and molecular mechanism underlying oil accumulation in developing castor seeds, we investigated the transcriptional profiles of the genes related to glycolysis, fatty acid biosynthesis, TAG assembly, and lipid droplet packaging in the developing seeds of cultivar ZB306. This analysis covered the five seed development stages, namely 10, 20, 35, 45, and 55 days after pollination (DAPs), which were designated as S1 to S5, respectively (Figure 1b; Table S1).

Using our previously generated RNA-seq data from EupDB (http://eupdb.liu-lab.com/, accessed on 20 November 2023), we compared the differential expression of gene transcripts between each of the five stages. The global expression of genes clearly exhibited a stage-specific pattern for each stage (Figure 1b). A total of 15,047 gene transcripts with FPKM (Fragments Per Kilobase Million) > 1 at one of the five seed stages were identified, of which 13,194 genes exhibited a differential expression at least at one stage of seed development (Figure S1). The Kyoto Encyclopedia of Genes and Genomes (KEGG) functional prediction revealed that these differentially expressed genes (DEGs) were predominantly involved in carbon metabolism, the biosynthesis of amino acids, and glycolysis/gluconeogenesis pathways (Figure S2). Furthermore, we conducted a hierarchical cluster analysis for the five stages using the FPKM values of all the differentially expressed genes (Table S2). As depicted in Figure 1b, these DGEs were classified into five clusters based on the K-means algorithm and were functionally associated with tissue development (GO: 0009888), RNA binding (GO: 0003723), cell wall biogenesis (GO: 0042546), ribosome biogenesis (GO: 0042254), and seed oil body biogenesis (GO: 0010344), respectively. In particular, the global expression of genes exhibited a stage-specific pattern of each stage (Figure 1b). For instance, a large number of genes involved in fatty acid metabolism (ko01212) and glycerolipid metabolism (ko00561) were highly expressed in Cluster5 (C5), which corresponds to the stage of rapid oil accumulation (Figure S3). On the other hand, genes clustered in C2 exhibited high expression in S1 and S2, and low expression in S4 and S5, and were enriched in glycolysis/gluconeogenesis (ko00010) and carbohydrate metabolism (ko01200) (Figure S4). The distinct gene expression patterns strongly implied that there were physiological and molecular differences at different stages of seed development.

### 2.2. Identification of Key Enzyme Genes Involved in the Production of Substrates for Fatty Acid Biosynthesis in the Glycolysis Pathway

**Figure 2:**
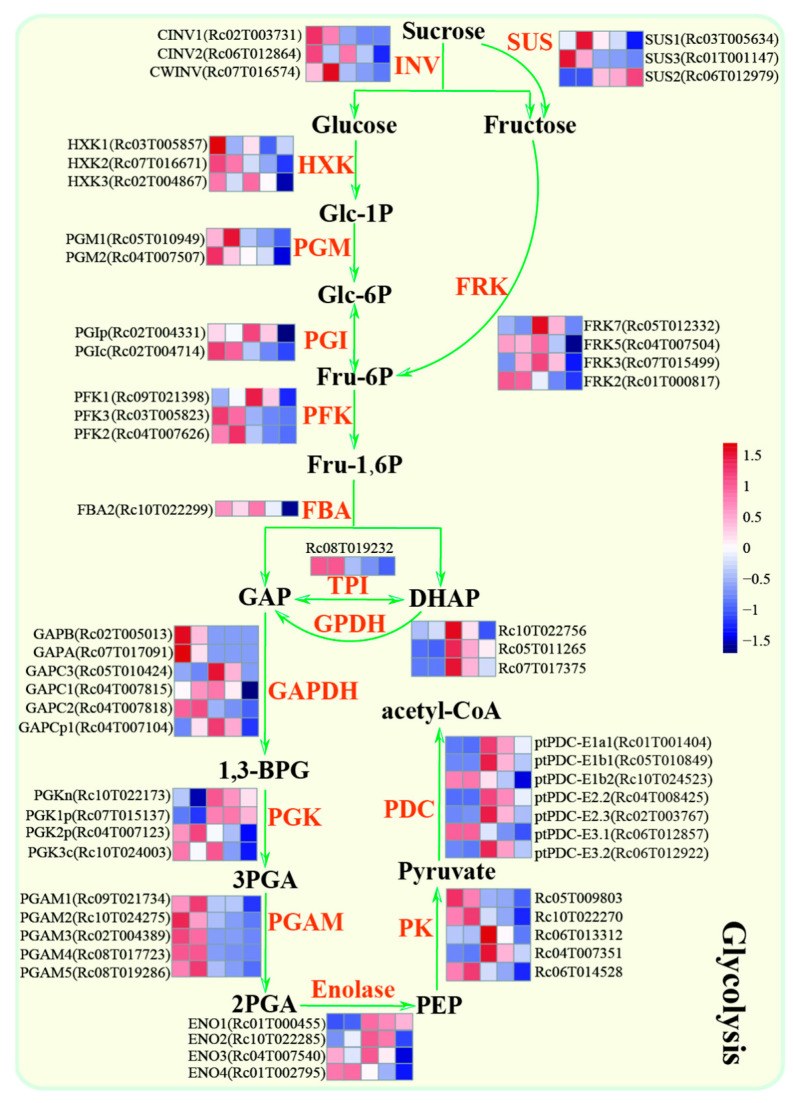
The schematic diagram of glycolysis biosynthetic pathways and the genes’ expression patterns during castor bean seed development. Gene expression changes at five developmental stages are expressed as log2-fold change. The red and blue colors represent up-regulated and down-regulated genes.

In typical heterotrophic seeds, where the energy and carbon resources for seed development and storage materials depend entirely on the sugars produced from photosynthesis in leaf tissues, the transportation of sugars from the leaves to the seeds is crucial for providing the necessary energy and carbon resources to drive seed metabolism and accumulate storage materials [19,20]. Sucrose, which is produced from photosynthesis in leaf tissues, serves as the primary source of energy and carbon resources. It is typically transported to developing seeds through sucrose transporters (SUTs) or sucrose carrier proteins (SUCs) [21]. Based on the castor bean reference genome, three homologous genes encoding SUTs were identified: *RcSUT1* (*Rc02T003776*), *RcSUT2* (*Rc01T000649*), and *RcSUT3* (*Rc02T004405*). These genes are responsible for the long-distance transportation of sucrose in the castor bean [19]. However, the functions of *RcSUT1*, *RcSUT2*, and *RcSUT3* are differentiated during sucrose transportation. Specifically, *RcSUT2* plays a primary role in transporting sucrose from the leaves to the developing seeds, providing the necessary energy and carbon resources to drive seed development and accumulate storage materials [19]. Generally, sucrose synthase (SUS) and invertase (INV) play critical roles in the initial stage of carbon metabolism, mainly involved in the interconversion between sucrose and glucose and/or fructose [22]. Similarly, we identified five *RcSUS* genes: *RcSUS1* (*Rc03T005634*), *RcSUS2* (*Rc06T012979*), *RcSUS3* (*Rc01T001147*), *RcSUS4* (*Rc04T008513*), and *RcSUS5* (*Rc05T012176*), as well as three *RcINVs* genes: *RcCINV1* (*Rc02T003731*), *RcCINV2* (*Rc06T012864*), and *RcCWINV* (*Rc07G016574*) (Figure 2). Upon examining the expression profiles of these *RcSUS* and *RcINV* genes in different tissues, we observed that only three *RcSUS* genes (*RcSUS1–3*) were highly expressed during seed development. *RcSUS1*, *RcSUS3*, and all *RcINV* genes were highly expressed in the early and/or middle stages of seed development (Table S3), while *RcSUS2* was highly expressed in the late stage of seed development (Table S4). Subsequently, glucose and fructose enter into the glycolysis reaction.

Glycolysis is a central process of energy transfer and carbon conversion that begins with glucose and ends with pyruvates. It provides carbon substrates for fatty acid synthesis [23]. As depicted in Figure 2, the key enzymes hexokinase (HXK), phosphoglucomutase (PGM), and phosphoglucose isomerase (PGI) are responsible for the successive conversion of glucose into glucose-1-phosphate (Glc-1P), glucose-6-phosphate (Glc-6P), and fructose-6-phosphate (Fru-6P) [24,25,26]. Additionally, fructokinases (FRK), phosphofructokinase (PFK), and fructose bisphosphate aldolase (FBA) are responsible for converting fructose into Fru-6P, Fru1,6P (fructose-1,6-bisphosphate), glyceraldehyde-3-phosphate (GAP), and/or dihydroxyacetone phosphate (DHAP), respectively [25,27,28]. The interconversion between DHAP and GAP is catalyzed by triosephosphate isomerase (TPI), while the conversion of DHAP into GAP is catalyzed by glycerol-3-phosphate dehydrogenase (GPDH) [29,30]. Subsequently, GAP is converted to 1,3-bisphosphoglycerate (1,3-BPG) by GAPDH (glyceraldehyde-3-phosphate dehydrogenase), which is a central enzyme in the glycolytic pathway. GAPDH is classified into three types: GAPA/B, GAPC, and GAPCp) [31]. Additionally, PGK (phosphoglycerate kinase), PGAM (phosphoglycerate mutase), enolase (ENO), and pyruvate kinase (PK) catalyze the conversion of 1,3-BPG into 3-phosphoglycerate (3PGA), 2PGA, phosphoenolpyruvate (PEP), and pyruvate [32,33,34]. Finally, pyruvate is converted to acetyl-coenzyme A (acetyl-CoA) by the pyruvate dehydrogenase complex (PDC) [35].

Based on the metabolic process, a total of 66 genes involved in the glycolysis pathway were identified in the castor genome. Among them, 34 genes exhibited higher expression levels during the early or middle stages of seed development, compared to the late stage. In particular, 58 genes were identified as DEGs between any two stages according to the thresholds of absolute log2-fold change ≥ 2 and q value ≤ 0.05. Most of these genes exhibited a significant difference between the early stage (S1 or S2) and the late stage (S5) (Table S3). As shown in Figure 2, most genes involved in glycolysis were highly expressed in the early stage. These genes include *RcHXK1* (*Rc03T005857*), *RcHXK2* (*Rc07T016671*), *RcFRK2* (*Rc01T000817*), *RcPGMs* (*Rc05T010949* and *Rc04T007507*), *RcPFK2* (*Rc04T007626*), *RcPFK3* (*Rc03T005823*), *RcGAPC2* (*Rc04T007818*), *RcPGK2p* (*Rc04T007123*), *RcPGAM2* (*Rc02T004389*), *RcPGAM4* (*Rc08T019286*), *RcPK2* (*Rc10T022270*), *RcPK5* (*Rc06T014528*), *RcPDC-E1b2* (*Rc10T024523*), and *RcPDC-E3.1* (*Rc06T012857*) (Table S4). Additionally, other highly expressed genes involved in glycolysis were identified in the middle stage of developing seeds. These genes include *RcHXK3* (*Rc02T004867*), *RcPGIp* (*Rc02T004331*), *RcPFK1* (*Rc09T021398*), *RcFBA2* (*Rc10T022299*), *RcENO2* (*Rc10T022285*), and three *GPDH* genes (*Rc10T022756*, *Rc05T011265*, and *Rc07T017375*) (Table S3). In conclusion, these highly expressed genes in the early and middle stages of seed development play a critical role in providing energy and substrates for fatty acid metabolism and lipid accumulation.

### 2.3. Identification of Key Rate-Limiting Enzyme Genes Involved in Fatty Acid and TAG Biosynthesis in Developing Castor Seeds

**Figure 3:**
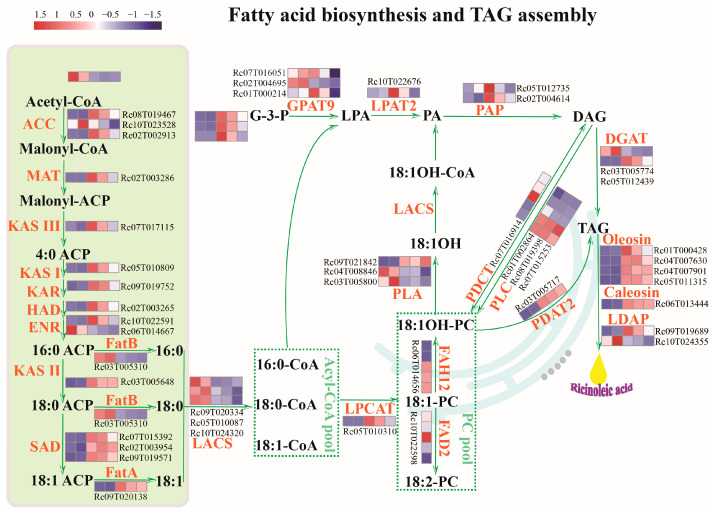
The schematic diagram of de novo fatty acid biosynthetic pathways in plastids and TAG assembly pathways in the endoplasmic reticulum (ER) and the genes’ expression patterns during castor bean seed development. Gene expression changes at five developmental stages are expressed as log2-fold change. The red and blue colors represent up-regulated and down-regulated genes.

As mentioned above, oil biosynthesis could be summarized into two main processes: fatty acid biosynthesis and TAG assembly [36]. While 82 genes directly involved in fatty acid biosynthesis and TAG assembly were identified based on the genome data (Table S3), the key rate-limiting enzyme genes and regulators in developing seeds are still largely unknown. Acetyl-CoA is the major substrate for fatty acid biosynthesis in the plastid through multiple carboxylations. This process involves acetyl-CoA carboxylase (ACCase) subunits, including *α-carboxyltransferase* (*α-CT*, *Rc10T023528*) and *ACC1* (*Rc02T002913*), as well as malonyl-CoA: acyl carrier protein *malonyltransferase* (*MAT*, *Rc02T003286*). The fatty acid elongase complex, which includes *beta-ketoacyl-acyl carrier protein (ACP) synthase III* (*KASIII*, *Rc05T011217*, *Rc07T017115*, and *Rc09T020453*), *KASI* (*Rc05T010809*), and *KASII* (*Rc03T005648*), is also involved in this process (Figure 3, Table S3). Based on the expression changes of genes at different stages of seed development, we identified 74 DEGs involved in fatty acid biosynthesis and TAG assembly (Table S3). Specifically, DEGs involved in fatty acid biosynthesis, such as *RcACCase* (*Rc09T019602* and *Rc10T023528*), *RcMAT* (*Rc02T003286*), *RcKASIII* (*Rc05T011217*), *RcKASI* (*Rc05T010809*), *RcKAR* (*Rc09T019752*), *RcHAD* (*Rc02T003265*), *RcKAR* (*Rc09T019752*), *RcKASII* (*Rc03T005648*), and *RcSAD* (*Rc09T019571* and *Rc02T003954*), are closely associated with oil accumulation during seed development (Figure 3). This strongly suggests that these genes may play a critical role in controlling oil biosynthesis in castor bean seeds.

The largest differences between ricinoleic acid and usual fatty acid biosynthesis in castor oils occur at the *sn*-2 position of the phosphatidylcholines (PC). During oil biosynthesis, the 18:1-PC (2-oleoyl-PC) is hydroxylated at the 12th carbon by *FAH12* (*fatty acid hydroxylase 12*, *Rc06T014656*), resulting in the synthesis of 18:1OH-PC (2-ricinoleoyl-PC) (Figure 3). The high levels of 18:1-OH in castor oils (TAGs) are due to the editing of 18:1OH-PC fatty acid out of PC and its incorporation into TAGs through acyl-CoA-dependent or -independent pathways [37]. In the assembly of TAGs, 18:1OH is released from 18:1OH-PC by *phospholipase A2* (*PLA_2_*, *Rc04T008846*, and *Rc03T005800*). It then enters into the acyl-CoA pool, where it is converted into 18:1OH-coenzyme A (CoA) and glycerol-3-phosphate (G3P). These compounds are further converted into lysophosphatidic acid (LPA), phosphatidic acid (PA), and diacylglycerol (DAG) through the successive actions of *glycerol-3-phosphate acyltransferase 9* (*GPAT9*, *Rc07T016051*, *Rc02T004695*, and *Rc01T000214*), *lysophosphatidic acid acyltransferase* (*LPAT*, *Rc10T022676*), and *PA phosphatase* (*PAP*, *Rc05T012735*, and *Rc02T004614*) [38] (Figure 3). Additionally, the choline head group is removed from PC to DAG through the actions of *phosphatidylcholine: diacylglycerol cholinephosphotransferase* (*PDCT*, *Rc07T016914*), *phospholipase C* (*PLC*, *Rc01T002864*, *Rc08T019398*, and *Rc07T015253*), or *phospholipase D* (*PLD*, *Rc03T005268*, *Rc03T006093*, and *Rc07T016897*), resulting in the production of PC-derived DAG [39]. Similarly, 18:1OH is removed from PC by PLA enzymes or by lysophosphatidylcholine acyltransferase (LPCAT) and transferred to CoA by reversible *LPCAT* (*Rc05T010310*) [39]. Finally, DAG is acylated at the sn-3 position by *diacylglycerol acyltransferase* (*DGAT*, *Rc03T005774*, and *Rc05T012439*), resulting in the synthesis of triacylglycerol (TAG) [40]. Additionally, *phospholipid: diacylglycerol acyltransferase* (*PDAT*, *Rc07T016532*, *Rc03T005717*, and *Rc07T015068*) catalyzes the biosynthesis of TAG by transferring a fatty acyl moiety from the sn-2 position of phospholipids to the sn-3 position of DAG. Once assembled, TAGs are stored in oil bodies that consist of structural proteins such as oleosin, caleosin, or steroleosin. These proteins are essential for maintaining the oil status and content in oilseeds. A total of 13 genes encoding proteins associated with oil bodies were identified (Table S4), including *Rc01T000428*, *Rc04T007630*, *Rc04T007901*, *Rc05T011315*, *Rc06T013444*, *Rc05T011647*, *Rc02T003557*, *Rc04T007735*, *Rc01T001091*, *Rc09T019689*, *Rc10T024355*, *Rc05T010729*, and *Rc09T019621*. The majority of these genes related to TAG assembly exhibited high expression levels during the middle and late stages of seed development (Figure 3).

### 2.4. Detection of The Transcription Factors Involved in Oil Accumulation

**Figure 4:**
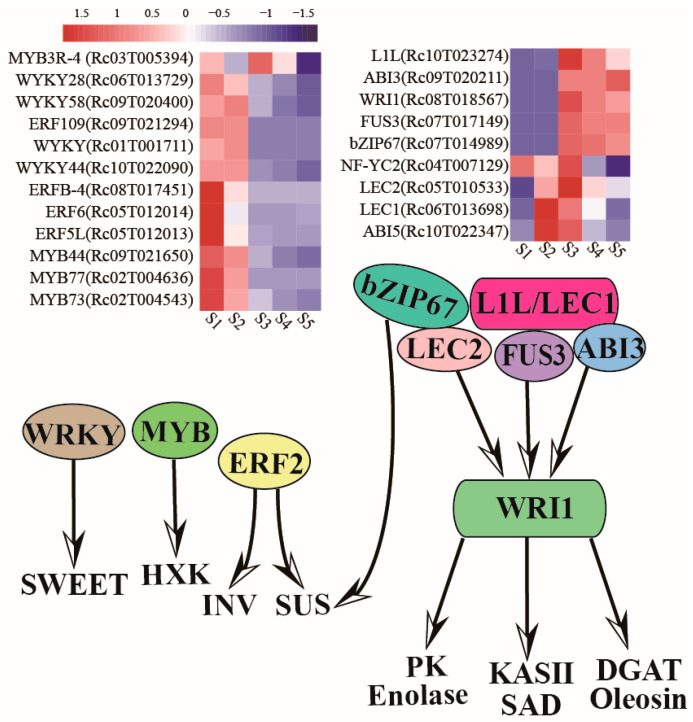
Representative transcription factors and genes regulate carbohydrate metabolism and fatty acid synthesis and their expression patterns during castor bean seed development. Gene expression changes at five developmental stages are expressed as log2-fold change. The red and blue colors represent up-regulated and down-regulated genes.

Transcription factors (TFs) play a crucial role in orchestrating seed development and TAG biosynthesis and storage in developing seeds. Various TFs, including MYB, WRKY, ERF/AP2, bZIP, and NF-Y, have been identified as critical regulators of seed development. Our analysis revealed that *RcWRKY44* (*Rc10T022090*), *RcWRKY58* (*Rc09T020400*), *RcMYB44* (*Rc09T021650*), *RcMYB73* (*Rc02T004543*), *RcERF72* (*Rc01T001153*), *RcERF74* (*Rc06T014906*), *RcbZIP18* (*Rc02T003449*), *RcbZIP29* (*Rc07T017285*), and *RcNF-YC1* (*Rc04T007762*) exhibited higher expression levels in the early stage of developing seeds compared to the middle or late stages (Figure 4, Table S4). This suggests that these TFs are likely involved in the regulation of castor seed development and energy metabolism. For instance, WRKY, MYB, and ERF may be involved in the regulation of gene expressions encoding *HXK*, *INV*, and *SUS*, respectively (Figure 4, Table S5). The master TFs involved in seed development, such as *Rc06T013698* encoding *LEC1*, *Rc05T010533* encoding *LEC2*, *Rc07T017149* encoding *FUS3*, *Rc09T020211* encoding *ABI3*, and *Rc08T018567* encoding *WRI1*, were found to be highly expressed and associated with seed development and oil accumulation (Table S4). This indicates that they play a crucial role in regulating castor seed development and oil accumulation in developing seeds. In particular, these *PK*, *enolase*, *KASII*, *SAD*, *DGAT*, and *Oleosin* genes may be directly or indirectly regulated by these master TFs, as shown in Figure 4.

### 2.5. Detection of The Expression Patterns of Oil Accumulation-Related Genes

**Figure 5:**
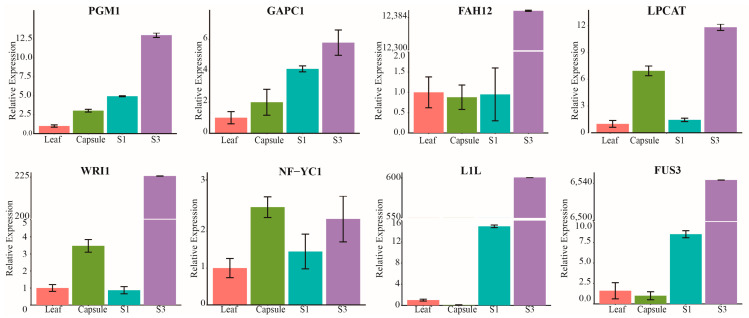
Relative expression levels of eight genes related to seed oil accumulation in castor by qRT-PCR analysis. The *x*-axis represents the leaf, capsule, early (S1), and middle (S3) development seeds. The *y*-axis represents the relative expression levels, which are the 2^−∆∆CT^ values of qRT-PCR compared to the reference gene. Data are shown as mean ± SD.

To experimentally validate the expression patterns of genes involved in seed oil accumulation in the castor, two glycolysis-related genes, two genes associated with TAG assembly, and four TFs involved in oil accumulation were chosen for the quantitative real-time polymerase chain reaction (qRT-PCR) and primers were listed in Table S6. As shown in Figure 5, seven of these genes exhibited the highest expression levels in the middle stage (S3) of castor seed development. In particular, the relative expression levels of *RcFAH12*, *RcWRI1*, *RcL1L*, and *RcFUS3* in S3 were significantly higher than in other periods or tissues, and their expression patterns measured by qRT-PCR were highly correlated with the FPKM values from transcriptomes (R = 1, *p* < 0.01, Figure S5). In addition, the expression pattern of *RcGAPC1* validated by qRT-PCR was positively correlated with the transcriptomic results (R = 0.99, *p* = 0.014, Figure S5). Hence, the expression levels of most genes/TFs involved in seed oil accumulation were high in the middle stage of seed development, and the results of RNA-seq were reliable.

## 3. Discussion

Based on the changes in oil accumulation at different stages of seed development, the genome data and global gene expression patterns in this study have provided a fundamental molecular profile of castor oil accumulation during seed development. This profile includes the processes related to sucrose supply, carbohydrate metabolism, fatty acid biosynthesis, and TAG assembly.

Sucrose transportation from the leaf (source) to the seed (sink) is crucial in non-photosynthetic oilseeds [41]. In this study, we identified *Rc01T000649*, which encodes *RcSUT2* and is preferentially expressed in developing seeds. This suggests that *RcSUT2* plays a specific role in sucrose transportation within castor-developing seeds. Since rapid oil accumulation occurs in the middle and late stages of seed development, it is expected that the essential genes involved in castor oil biosynthesis will be highly expressed during this stage. By analyzing the differential expression of the genes related to lipid biosynthesis, we identified a total of 151 critical genes involved in glycolysis, fatty acid biosynthesis, and TAG assembly pathways. In carbohydrate metabolism, before sugar enters glycolysis, we identified three *RcINVs* genes (*Rc02T003731*, *Rc06T012864*, and *Rc07T016574*) that were responsible for sucrose breakdown and ovule initiation during the early stage of castor seed development. Previous studies have also reported the critical role of *INV* genes in controlling early seed development and sugar metabolism in rice, maize, and cotton [42,43,44]. This suggests that *INV* genes are essential for regulating early seed development and physiological metabolism in plants.

Glycolysis is a crucial metabolic pathway for supplying precursors for oil biosynthesis [45]. In various oilseed crops such as Arabidopsis, sunflower, and oil palm, several limiting-rate enzyme genes involved in carbon allocation into oil have been identified to play a critical role in generating precursors for oil biosynthesis and regulating oil accumulation in developing seeds [30,33,46,47,48]. In our analysis, we identified several candidate genes (such as *RcGPDH*, *RcGAPC*, *RcPGK*, and *RcPGM*) in glycolysis that may act as limiting-rate enzyme genes for generating precursors of oil biosynthesis in castor-developing seeds. Fatty acid biosynthesis not only determines the composition of fatty acids in storage oils but also directly affects the oil content of seeds. We identified multiple candidate genes (such as *RcMAT*, *RcKAR*, *RcENR1*, *RcKASII*, and *RcSAD3*) encoding key enzymes responsible for fatty acid biosynthesis, including carbon chain elongation and the desaturation of fatty acids. Unlike other oilseed crops such as rapeseed, sunflower, and groundnut, castor seeds accumulate unique hydroxy fatty acids. Previous studies have found that the *FAH12* gene is responsible for the biosynthesis of ricinoleic acid in castor seeds [49]. However, transgenic Arabidopsis plants expressing *RcFAH12* were unable to accumulate a large amount of ricinoleic acid [50]. These results suggest that there is an efficient mechanism for the removal of ricinoleic acid synthesized in phospholipids and its transfer to TAGs in castor seeds, which is in contrast to Arabidopsis seeds. We also identified several key genes related to TAG assembly, such as *RcLPCAT*, *RcPDCT*, and *RcDGAT2*. These genes are involved in the entry of fatty acids into PC via the “acyl-editing” process, the transfer of an acyl group from PC to DAG, and the conversion of DAG into TAG [51,52,53]. In particular, the biosynthesis of unusual ricinoleic acid oils involves an independent phospholipid-diacylglycerol acyltransferase (PDAT) pathway during TAG assembly in castor-developing seeds [54]. The castor bean is often considered a model system for studying the molecular basis of oil accumulation in developing seeds. The accumulation of high amounts of ricinoleic acid-rich TAGs in castor seeds is attributed to both acyl-CoA-dependent and acyl-CoA-independent pathways.

So far, several transcriptional master regulators related to storage oil biosynthesis have been extensively studied, including MYB, ERF, WRI1, LEC1, and ABI3. These regulators are considered essential genes in the control of sugar metabolism and oil accumulation. WRKY, MYB, and ERF TFs could control carbon partitioning via regulation of sugar transporters, sucrose accumulation, and sucrose metabolism, respectively [55,56,57]. These TFs are highly expressed in the early and middle stages of castor seed development. In particular, TFs that control fatty acid biosynthesis are physiologically coupled with seed development in higher plants. These include NF-Ys genes, LEC1, LEC1-LIKE (L1L), NF-YC1, B3 domain TFs, LEC2, FUS3, and ABI3 [58,59,60,61,62,63]. As master regulators of seed development, LEC1, LEC2, FUS3, and ABI3 could directly induce the APETALA2 (AP2)-type TF WRI1 [63]. In this study, these TFs involved in oil accumulation were highly expressed in the middle and late stages of castor seed development. Furthermore, the expression levels of *RcNF-YC1* (*Rc04T007762*), *RcNF-YC2* (*Rc04G007129*), *RcERF72* (*Rc01T001153*), and *RcERF74* (*Rc06T014906*) were higher in the early stage of castor seeds. NF-Ys play essential roles in endosperm development by interacting with ERF TFs in monocots [64]. These results suggest that the large endosperm formation and fatty acid accumulation in the castor might be related to the preferential expression of NF-Ys. The regulatory mechanism of seed oil accumulation is complex and requires the coordination of multiple genes and TFs. Many studies have been unable to increase oil production due to the lack of sufficient substrates. Therefore, understanding the regulatory mechanisms of seed oil accumulation is crucial. This study is the first to identify all the candidate genes and TFs involved in carbohydrate metabolism, fatty acid biosynthesis, and TAG assembly in castor beans.

## 4. Materials and Methods

### 4.1. Plant Material

Castor seeds of the elite inbred line ZB306 were germinated in an incubator at 30 °C for two weeks. Subsequently, the seedlings were carefully transferred to the greenhouse of Southwest Forestry University, which was maintained at 30/25 °C day/night temperature with 16-h photoperiod until developing seeds were harvested. The developing seeds were sampled 10 days after pollination (DAP), 20 DAP, 35 DAP, 45 DAP, and 55 DAP, respectively. After removing the capsules, the seeds were immediately frozen in liquid nitrogen and stored at −80 °C until total RNA extraction. The seed oil of each sample was extracted and the content was measured using the previously described method [65].

### 4.2. Transcriptome Data Analysis

The gene expression levels for castor bean-developing ZB306 seeds were based on the raw sequenced reads of Illumina HiSeqTM 2000 platform, which contained five distinct seed development stages from set to ripening (10, 20, 35, 45, and 55 DAP) [17]. The BioProject accession of the above samples in NCBI was PRJNA787114, and quality control was performed using fastp for reads in the FASTQ format [66]. Subsequently, the clean reads were mapped to the newly published castor reference genome using HISAT2 (Hierarchical Indexing for Spliced Alignment of Transcripts 2) [15,67]. The SAM files were sorted and indexed using samtools [68]. The quantification of gene expression levels was performed by StringTie and differentially expressed genes (DEGs) were identified through edgeR software (version 4.0.3) with q value ≤ 0.05 and |log2fold change| ≥ 2 [69,70]. Additionally, functional annotations were obtained with eggNOG mapper v6.0, including the COG, GO, and KEGG [71]. A hierarchical clustering analysis was conducted to elucidate the gene expression patterns across all five seed developmental stages. This analysis was visualized using the R package ClusterGVis (https://github.com/junjunlab/ClusterGVis, version 0.0.2, accessed on 9 June 2023). Furthermore, GO (Gene Ontology) functional enrichment analysis of these genes in each cluster was performed using the clusterProfiler (version 4.10.0) [72].

### 4.3. Identification of The Genes Related to Oil Accumulation in The Castor Genome

Firstly, protein sequences of the newly published castor bean genome were used to build a personal castor database. Then, amino acid sequences of genes related to oil accumulation were retrieved from the website of ARALIP (ARABIDOPSIS ACYL-LIPID METABOLISM, http://aralip.plantbiology.msu.edu/locations, accessed on 9 February 2023), other species genome, and the first version castor bean reference genome. Utilizing these sequences as queries, we conducted a BLAST (basic local alignment search tool) program against our local castor database with the E-value < 1E-5. The blast results were then subjected to rigorous filtering criteria. First, only the longest transcript of each gene was selected to represent the gene locus, ensuring a comprehensive representation of each gene. Secondly, genes with alignment length of less than 100 amino acids were excluded. Lastly, we discarded any alignments that had identity percentages below 30%, further refining the quality of our data. To precisely identify all members of specific gene families in the newly published genome of castor bean, the phylogenetic analyses were conducted using Muscle (v5), IQ-TREE (v1.6.12), and iTOL (https://itol.embl.de, accessed on 16 February 2023) software [73,74].

### 4.4. Promoter Analysis and Subcellular Localization Prediction

To further predict the regulation relations between fatty acid-related genes and core transcription factors, cis-elements in the gene promoter sequences were detected through the website of New PLACE (https://www.dna.affrc.go.jp/PLACE/?action=newplace, accessed on 29 August 2023) [75]. To better establish the functions of genes located in different subcellular compartments, we predicted the genes’ subcellular localization via online websites Cell-PLoc 2.0 (http://www.csbio.sjtu.edu.cn/bioinf/Cell-PLoc-2/, accessed on 10 September 2023) and WoLF PSORT (https://wolfpsort.hgc.jp/, accessed on 10 September 2023).

### 4.5. Gene Expression Pattern Analysis

To examine the genes’ tissue-specific expression patterns and the expression intensity during castor bean seed development process, the pheatmap package within the R software (version 1.0.12) environment was utilized to visualize the levels of gene expression. The expression value for each gene was represented as the average expression levels of three biological replicates.

### 4.6. qRT-PCR Validation

A total of 8 genes were selected to validate their expression patterns in leaf, capsules, and developing seeds in the early (S1) and middle stages (S3). The total RNAs were extracted and then reverse-transcribed to complementary DNA (cDNA) according to the method mentioned above [17]. qRT-PCR amplifications were carried out using the PerfectStart Green qPCR SuperMix kit (TransGen Biotech, Beijing, China). Three biological replicates with three technical replicates were performed for each gene and each sample. The relative expression levels of each gene were mean ± SD, employing ACTIN as reference gene. The primers used in this study were picked using Primer 3 web (v. 0.4.0, https://bioinfo.ut.ee/primer3-0.4.0/, accessed on 29 November 2023).

## Data Availability

Data is contained within the article and Supplementary Materials.

## Supplementary Materials

The following supporting information can be downloaded at https://www.mdpi.com/article/10.3390/ijms25010092/s1.
