# Supplementary material for: Functional Genome Analyses Reveal the Molecular Basis of Oil Accumulation in Developing Seeds of Castor Beans
Source: Int J Mol Sci. 2023 Dec 20;25(1):92. doi: 10.3390/ijms25010092 (PMC10778879; doi:10.3390/ijms25010092)
Supplement: Supplementary file 1 [file ijms-25-00092-s001.zip › ijms-2759544-supplementary.pdf]

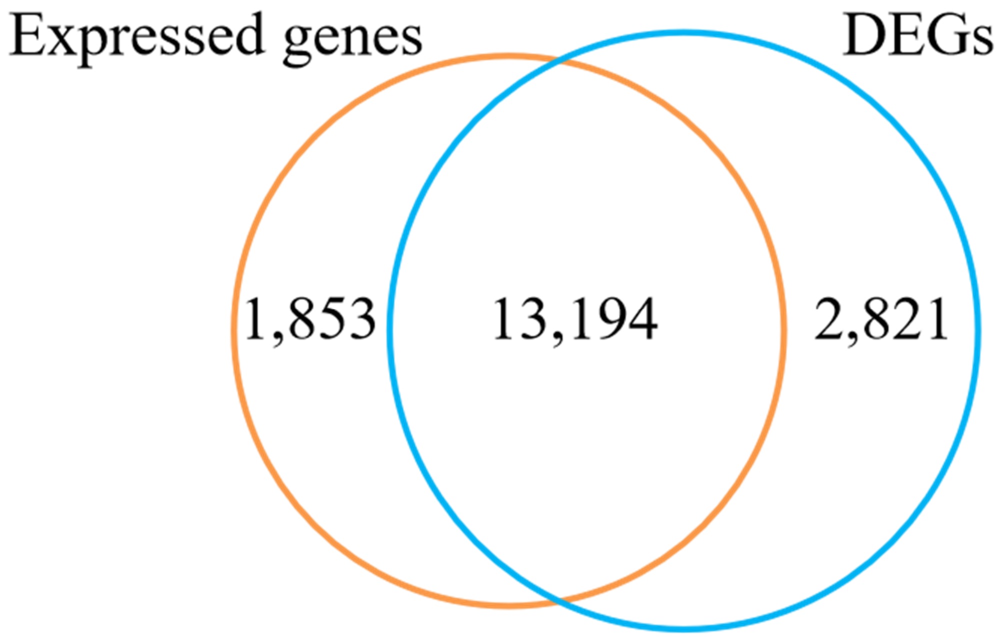

Figure S1. Overlap genes between DEGs and expressed genes.

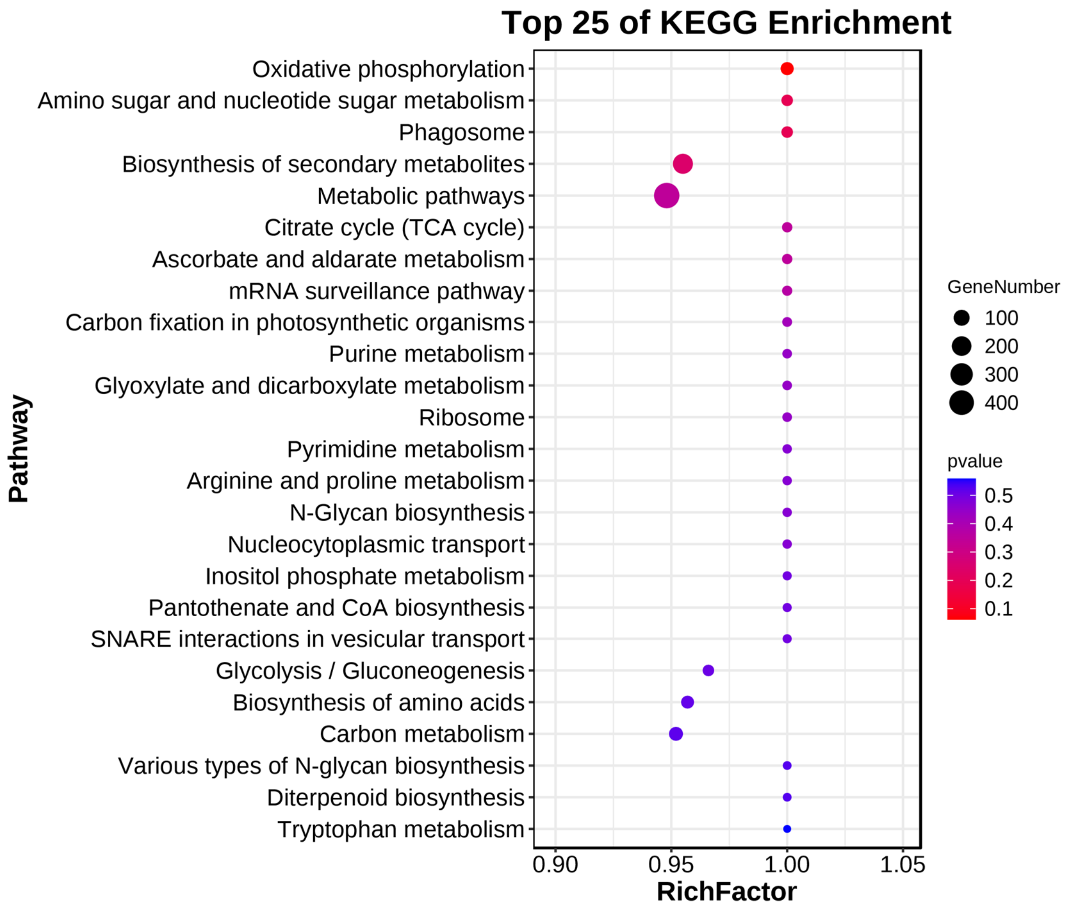

Figure S2. KEGG enrichment analysis of 13,194 differently expressed genes.

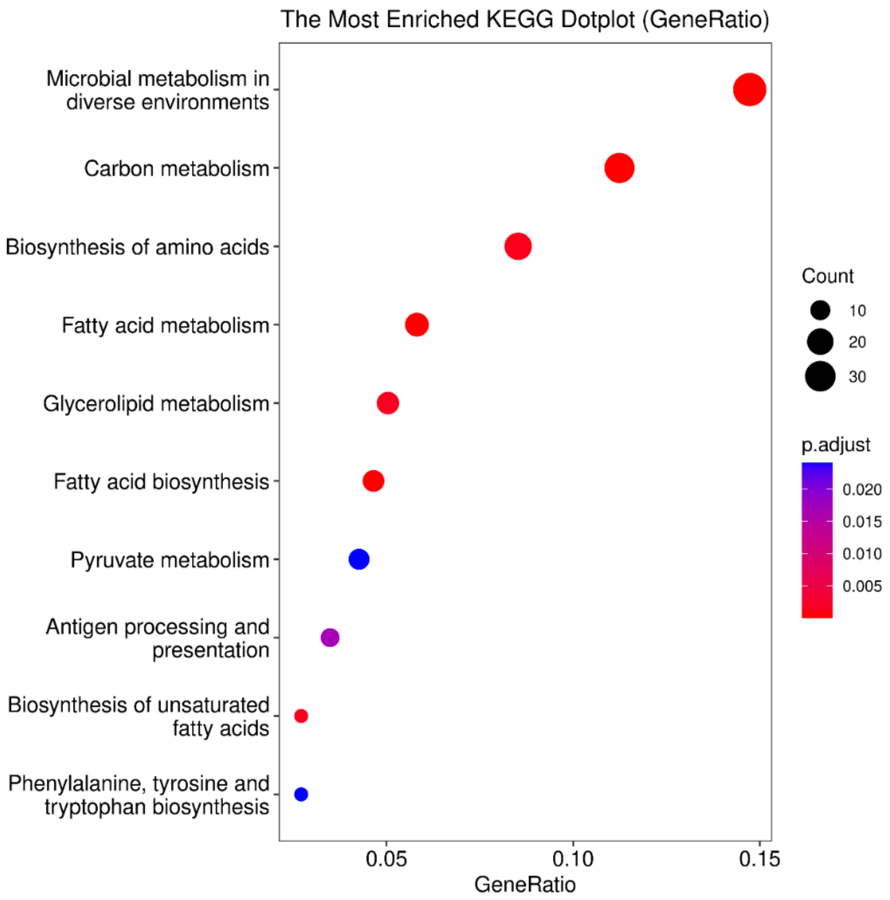

Figure S3. KEGG enrichment analysis of genes clustered in C5.

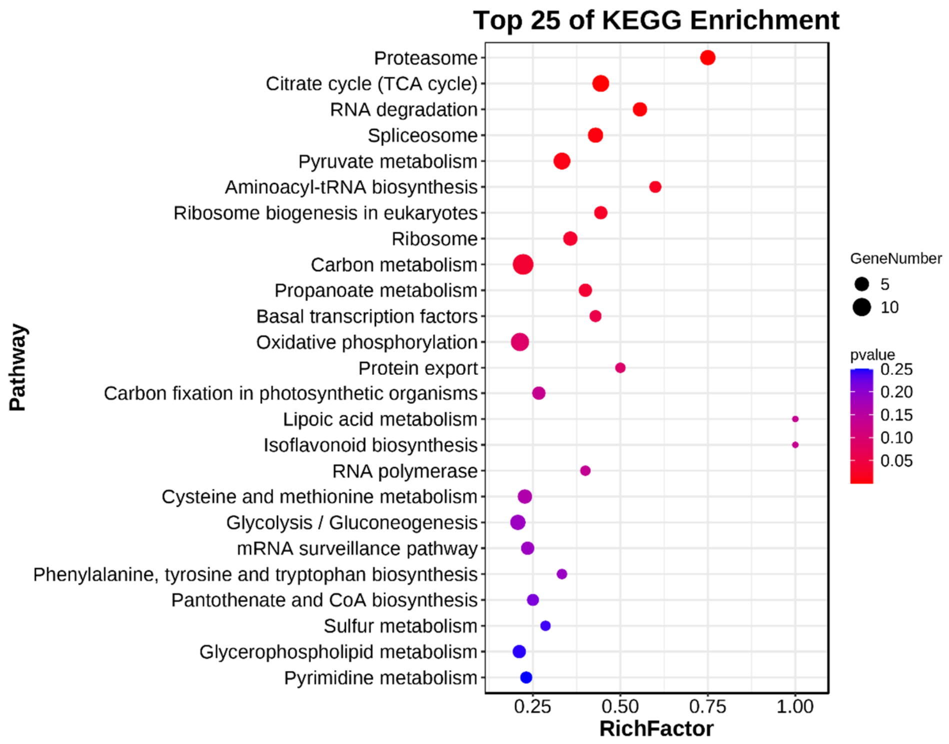

Figure S4. KEGG enrichment analysis of genes clustered in C2.

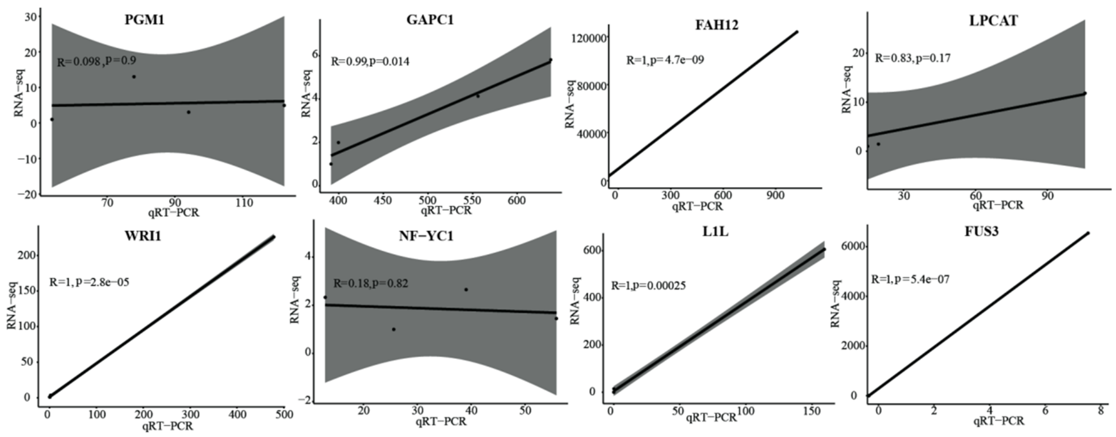

Figure S5. Correlation analysis of 8 genes involved in seed oil accumulation between RT-qPCR and RNA-Seq.
